# Hemoplasma Infection in HIV-positive Patient, Brazil

**DOI:** 10.3201/eid1412.080964

**Published:** 2008-12

**Authors:** Andrea Pires dos Santos, Rodrigo Pires dos Santos, Alexander W. Biondo, José M. Dora, Luciano Z. Goldani, Simone Tostes de Oliveira, Ana Maárcia de Sá Guimarães, Jorge Timenetsky, Helio Autran de Morais, Félix H.D. González, Joanne B. Messick

**Affiliations:** Universidade Federal do Rio Grande do Sul, Porto Alegre, Rio Grande do Sul, Brazil (A.P. Santos, R.P. Santos, J.M. Dora, L. Z. Goldani, S.T. Oliveira, F.H.D. González); Universidade Federal do Paraná, Curitiba, Paraná, Brazil (A.W. Biondo); University of Illinois, Urbana, Illinois, USA (A.W. Biondo); Universidade de São Paulo, São Paulo, Brazil (A.M. de Sá Guimarães, J. Timenetsky); University of Wisconsin, Madison, Wisconsin, USA (H.A. de Morais); Purdue University, West Lafayette, Indiana, USA (J.B. Messick); 1 These authors contributed equally to this article.

**Keywords:** AIDS, Bartonella, Haemobartonella, HIV, Mycoplasma, Brazil, dispatch

## Abstract

Hemotrophic mycoplasmas infect a variety of mammals. Although infection in humans is rarely reported, an association with an immunocompromised state has been suggested. We report a case of a *Mycoplasma haemofelis*–like infection in an HIV-positive patient co-infected with *Bartonella henselae*.

Hemoplasmas are a group of bacteria that infect animals. They are small epicellular parasites that adhere to the host’s erythrocytes. Diseases caused by these bacteria range from acute hemolytic anemia to asymptomatic infection. It is generally thought that most *Mycoplasma* spp. are host specific. However, there are occasional reports of infection in an animal species not perceived as primary hosts. These infections may have a pathologic effect, particularly when predisposing conditions, such as immunodeficiency, are present (*1*). We report a case of *Mycoplasma haemofelis*–like infection in an HIV-positive patient with disseminated *Bartonella henselae* infection.

## The Study

A 34-year-old HIV-positive man was hospitalized in Brazil in September 2006 with a 30-day history of night sweats; loss of appetite; productive cough; muscle pain; and cervical, axillary, and inguinal lymphadenomegaly. Results of pulmonary, cardiovascular, and abdominal examinations were normal. Abnormal lymph nodes were 2 cm in diameter, firm, and not tender. The patient owned 5 cats and showed signs of multiple cat scratches and bites on his hands and arms. He had received an HIV-positive diagnosis 5 years earlier.

At admission, his CD4 cell count was 286 cells/mm^3^ (reference range 500–1,500 cells/mm^3^), and viral load was 38,100 copies/mL. Additional laboratory findings included anemia, hematocrit 29% (reference range 38%–50%); leukopenia, 3,300 leukocytes/μL (4,300–10,000 leukocytes/μL); thrombocytopenia, 108,000 platelets/µL (150,000–450,000 platelets/μL); aspartate aminotransferase 66 U/L (15–40 U/L), alanine aminotransferase 79 U/L (10–40 U/L), and lactate dehydrogenase 657 U/L (240–480 U/L). Blood cultures yielded no bacterial growth; sputum cultures were negative for acid-fast bacilli, bacteria, and fungi. Test results were negative for hepatitis B and C, human T lymphotropic virus type 1, syphilis, chlamydia, and cryptococcus infections. Bone marrow and lymph node biopsy and culture results were negative for mycobacterial or fungal infections.

Abdominal computed tomography showed hepatomegaly, splenomegaly, and hypoechoic lesions on the spleen. Transesophageal echocardiography showed no lesions compatible with infective endocarditis. An inguinal node biopsy showed granuloma with necrosis suggesting cat-scratch disease, and no signs of acid-fast bacilli or fungi. Many bacilli suggestive of *Bartonella* spp. were observed by Warthin-Starry staining, and antibodies against *B*. *henselae* (titer 256) were detected in serum. Treatment with doxycycline was initiated and symptoms subsequently subsided. The patient was discharged and instructed to continue antiretroviral therapy and oral doxycycline.

In June 2007, the patient was hospitalized with fever, malaise, weight loss, and lymphadenomegaly. Echocardiography showed mitral vegetations. Multiple hepatic hypoechoic lesions were found by abdominal computed tomography. Lymph node biopsy specimens showed tiny bacilli by Warthin-Starry staining. The patient had prematurely discontinued antiretroviral and antimicrobial drug treatment, which may have predisposed him to endocarditis and hepatic peliosis. He was treated with doxycycline and gentamicin for *Bartonella* spp. infection. His symptoms disappeared and an echocardiogram 17 days later showed resolution of mitral vegetations. He was discharged and instructed to continue antiretroviral therapy and oral doxycycline for *Bartonella* spp. infection. Ten months after discharge, the patient returned for a follow-up visit while taking recommended therapy. He had no clinical signs and his laboratory findings were improved.

During the patient’s first hospitalization in 2006, blood was collected into tubes containing EDTA and 2 aliquots of DNA were extracted (DNeasy Blood and Tissue Kit; QIAGEN, Valencia, CA, USA) at the Hospital de Clínicas de Porto Alegre. DNA was tested by PCR for *Bartonella* spp. infection (*2*) and by additional PCR protocols for feline hemoplasmas, including *Mycoplasma haemofelis* (*3*), “Candidatus *M*. *haemominutum*” (*4*), and “Candidatus *M*. *turicensis*” (A.P. Santos, unpub. data). These bacteria infect cats and possible infection of this patient was investigated. Positive controls for *M*. *haemofeli*s included DNA extracted from naturally (GenBank accession no. EU930823) and experimentally infected cats (*3*). Three positive controls for *B*. *henselae* (type 1, type 2, and Houston strain) were used. Negative controls included ultrapure water and DNA extracted from blood of a healthy person and a noninfected cat. All negative controls were negative by PCR.

Amplicons of the expected size were obtained in *Bartonella* spp. and *M*. *haemofelis* PCRs. The 393-bp PCR product for *M*. *haemofelis* (Figure) was purified (Zymoclean Gel DNA Recovery Kit; Zymo Research, Orange, CA, USA), cloned (pGEM-T EasyVector; (Promega, Madison, WI, USA), and sequenced (Purdue Genomics Core Facility, West Lafayette, IN, USA). The fragment was 99% homologous with *M*. *haemofelis* 16S rDNA gene sequences in the GenBank database. To assess the sequence of the 16S rRNA gene, we designed species-specific primers based on the *M*. *haemofelis* sequence (forward primer 5′-ATG CAA GTC GAA CGG ATC TT-3′; reverse primer 5′-TCC AAT CAG AAT GTT CAC TC-3′). PCR product amplified from the patient’s blood was purified and sequenced. A 1,214-bp sequence was submitted to GenBank (accession no. EU888930); it was 99% homologous with the sequence for *M*. *haemofelis*.

**Figure:**
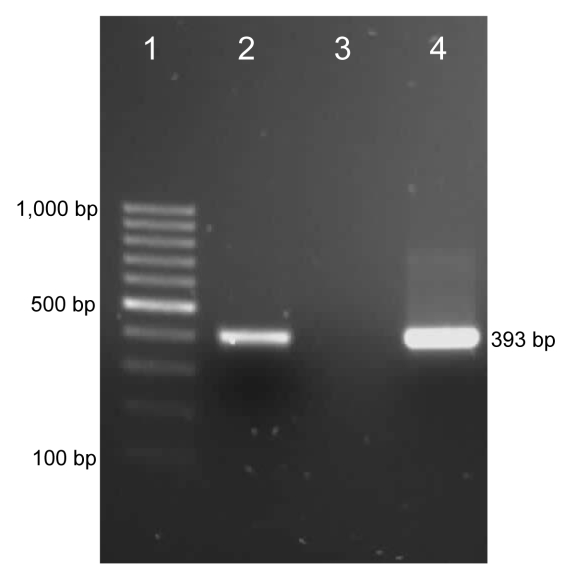
PCR results for detection of a *Mycoplasma haemofelis*–like organism in an HIV-positive patient. Lane 1, 100-bp marker; lane 2, positive control (DNA from blood of an *M*. *haemofelis*–positive cat); lane 3, negative control (water); lane 4, DNA from blood of the patient.

The possibility that the patient’s cats might be involved in zoonotic transmission was also investigated. Two weeks after the patient’s first blood collection, blood was collected from the 5 cats, and DNA was extracted at the Veterinary Hospital of the Universidade Federal do Rio Grande do Sul, Porto Alegre. Two of the cats were positive by PCR for *M*. *haemofelis* and all 5 cats were positive for *Bartonella* spp. The cats were not tested for other infectious agents.

PCRs for hemoplasmas were performed in duplicate at 2 laboratories (Universidade de São Paulo, São Paulo, Brazil and Purdue University, West Lafayette, IN, USA) by using split aliquots. PCR results were reproducible. During the patient’s second hospitalization, the same PCRs were used and the patient was positive for *Bartonella* spp. but negative for hemoplasmas.

## Conclusions

Hemoplasma infections may occur more frequently than is generally recognized, given that these organisms fail to grow in culture and only a few laboratories are equipped to detect and identify hemoplasmas (*1*). Disease associations with latent mycoplasma infections in immunocompromised and nonimmunocompromised patients are now emerging. Increasing numbers of human patients with compromised immune systems living near cats increases the possibility that hemoplasma infections may also emerge in this population.

There are no molecular studies to date documenting hemoplasma infection in humans. However, it has been suggested that such infections may be seen in immunocompromised patients (*5*). A hemotrophic mycoplasma infection was reported in patients with systemic lupus erythematosus (SLE) (*6*). A 417-bp sequence detected in 1 SLE patient also showed 99% homology with *M*. *haemofelis* (*7*). Sequence data from another hemotrophic mycoplasma infection in an anemic human patient were reported in GenBank. However, the sequence of 178 bp of the 16S rRNA gene (accession no. EU014880) was more closely related to *M*. *suis* and *M*. *wenyonii* (96%–100%) and only 75% homologous to *M*. *haemofelis* (*8*).

Epidemiologic studies have linked cat bites and scratches and flea-infested cats with transmission of *B*. *henselae* to humans (*9*). *B*. *henselae* and *B*. *quintana* are causative agents of bacillary angiomatosis, bacillary peliosis, and cat-scratch disease in humans. Peliosis hepatis and lymph node angiomatosis, as seen in this patient, have been associated with *B*. *henselae* infection (*10*). *M*. *haemofelis* DNA has also been detected in cat fleas (*Ctenocephalides felis*); *C*. *felis* may be involved in transmission of *M*. *haemofelis* among cats (*11*). Additional studies have documented experimental transmission by administration of infected blood intravenously, intraperitoneally, and orally. Hemoplasma DNA is present in saliva and feces of cats, which suggests that aggressive interactions among cats involving biting may lead to transmission of the organism (*12*). To our knowledge, there is only 1 other report that cats can be co-infected with *M*. *haemofelis* and *B*. *henselae* (*13*).

As with other *Mycoplasma* spp., hemoplasmas might act as a cofactor in HIV infection, contributing to acceleration of the course of the disease (*14*). Further studies are needed to establish the role and prevalence of hemoplasma infection in AIDS patients, as well as the zoonotic potential of *M*. *haemofelis*.
